# The global stock of research evidence relevant to health systems policymaking

**DOI:** 10.1186/1478-4505-11-32

**Published:** 2013-09-04

**Authors:** Michael G Wilson, Kaelan A Moat, John N Lavis

**Affiliations:** 1 McMaster Health Forum, McMaster University, 1280 Main St. West, MML-417, Hamilton, ON, Canada L8S 4L6, USA; 2 Centre for Health Economics and Policy Analysis, McMaster University, 1280 Main St. West, CRL 282 Hamilton, ON, Canada L8S 4K1, USA; 3 Department of Clinical Epidemiology and Biostatistics, McMaster University, MUMC 2C1, Hamilton, ON, Canada L8S 4K1, USA; 4 Health Policy PhD Program, McMaster University, 1280 Main St. West, CRL 209, Hamilton, ON, Canada L8S 4K1, USA; 5 Department of Political Science, McMaster University, 1280 Main St. West, KTH 527, Hamilton, ON, Canada L8S 4M4, USA; 6 Department of Global Health and Population, Harvard School of Public Health, 665 Huntington Ave, Building 1, Room 1104, Boston, MA 02115, USA

## Abstract

**Background:**

Policymakers and stakeholders need immediate access to many types of research evidence to make informed decisions about the full range of questions that may arise regarding health systems.

**Methods:**

We examined all types of research evidence about governance, financial and delivery arrangements, and implementation strategies within health systems contained in Health Systems Evidence (HSE) (http://www.healthsystemsevidence.org). The research evidence types include evidence briefs for policy, overviews of systematic reviews, systematic reviews of effects, systematic reviews addressing other questions, systematic reviews in progress, systematic reviews being planned, economic evaluations, and health reform and health system descriptions. Specifically, we describe their distribution across health system topics and domains, trends in their production over time, availability of supplemental content in various languages, and the extent to which they focus on low- and middle-income countries (LMICs), as well as (for systematic reviews) their methodological quality and the availability of user-friendly summaries.

**Results:**

As of July 2013, HSE contained 2,629 systematic reviews of effects (of which 501 are Cochrane reviews), 614 systematic reviews addressing other questions, 283 systematic reviews in progress, 186 systematic reviews being planned, 140 review-derived products (evidence briefs and overviews of systematic reviews), 1,669 economic evaluations, 1,092 health reform descriptions, and 209 health system descriptions. Most systematic reviews address topics related to delivery arrangements (n = 2,663) or implementation strategies (n = 1,653) with far fewer addressing financial (n = 241) or governance arrangements (n = 231). In addition, 2,928 systematic reviews have been quality appraised with moderate AMSTAR ratings found for reviews addressing governance (5.6/11), financial (5.9/11), and delivery (6.3/11) arrangements and implementation strategies (6.5/11); 1,075 systematic reviews have no independently produced user-friendly summary and only 737 systematic reviews have an LMIC focus. Literature searches for half of the systematic reviews (n = 1,584, 49%) were conducted within the last five years.

**Conclusions:**

Greater effort needs to focus on assessing whether the current distribution of systematic reviews corresponds to policymakers’ and stakeholders’ priorities, updating systematic reviews, increasing the quality of systematic reviews, and focusing on LMICs.

## 

“Congress has provided vital funding for research that compares the effectiveness of different treatments, and this should help reduce uncertainty about which treatments are best. But we also need to fund research that compares the effectiveness of different systems of care – to reduce our uncertainty about which systems work best for communities. These are empirical, not ideological questions”. Atul Gawande, The New Yorker, 1 June 2009, p. 44

## Background

Policymakers and stakeholders need immediate access to many types of research evidence to make informed decisions about the full range of questions they may have regarding health system arrangements and implementation strategies (with the latter including those aimed at supporting the use of research evidence at the level of citizens, providers, organizations, and policymakers). While policymakers need to consider many factors for any given decision about health systems (e.g., institutional constraints, interests of stakeholders affected by decisions, and the values and preferences of the public), research evidence can also help support and inform their efforts to strengthen or reform health systems or get cost-effective programs, services, and drugs to those who need them. Atul Gawande got it mostly right: “these are empirical, not *just* ideological questions”.

The timeliness of research evidence was one of two factors that emerged with some consistency in a systematic review of the factors that increased the prospects for research use in policymaking [1]. When the research literature has already been identified, selected, appraised and synthesized in a systematic and transparent way, health system policymakers can move directly to assessing how much confidence they can place in the review (i.e., its quality), the local applicability of the review’s findings, and what the findings mean for their setting [2]. Stakeholders, such as professional associations and citizen groups, also need timely access to many types of research evidence to inform their advocacy efforts focused on health systems. Researchers and research funding agencies need systematic reviews to identify gaps in knowledge about health systems (both primary studies and systematic reviews) and domains that could benefit from overviews of systematic reviews, as well as to put the findings of any new health systems research in the context of existing research [1].

Questions about the comparative effectiveness of one health system arrangement over another (such as using nurses rather than doctors to deliver certain forms of care) are one of the types of questions for which policymakers and stakeholders may turn to systematic reviews [3]. The likelihood of them being misled by research evidence about comparative effectiveness is lower and confidence in what effects they can expect from a health system arrangement is higher with a systematic review than with an individual study [4]. However, what the quotation from Atul Gawande fails to point out is that policymakers and stakeholders can also turn to systematic reviews to address questions best answered using qualitative and mixed-methods studies, such as questions regarding patients’ views about and experiences with problems encountered in health systems and with options for addressing these problems [5]. Furthermore, given often constrained resources, policymakers also need to consider value for money in any decision they make, which requires access to locally applicable economic evaluations about the various policy levers at their disposal. To further support their decisions, policymakers may also wish to turn to descriptions of health reforms undertaken in other jurisdictions to better understand what was done, how, and why, as well as to descriptions of other health systems to help determine the local applicability of research evidence generated in these systems to their own.

Policymakers and stakeholders are relatively well served by existing databases such as Medline (particularly when validated search strategies are employed) [6,7] and the Cochrane Library (which contains both the Cochrane Database of Systematic Reviews and the Database of Reviews of Effects) if their questions about comparative effectiveness pertain to clinical programs and services or to drugs [3]. They are also well served by Health Evidence™ (http://www.healthevidence.org) if their questions about comparative effectiveness pertain to public health programs and services [8]. More recently, if their questions pertain to the governance, financial and delivery arrangements within which programs, services and drugs are provided, and to implementation strategies for these programs, services and drugs, they are well served by Health Systems Evidence (HSE) (http://www.healthsystemsevidence.org). HSE provides a comprehensive inventory of nine types of research evidence, namely evidence briefs for policy (i.e., a document that summarizes how the findings from a number of systematic reviews pertain to a pressing problem, select options for addressing the problem, and key implementation considerations), overviews of systematic reviews, systematic reviews of effects, systematic reviews addressing other types of questions, systematic reviews in progress, systematic reviews being planned, economic evaluations, health reform descriptions, and health system descriptions [9,10].

All of these existing databases are further strengthened by significant efforts from a large and growing number of groups to package, quality appraise, and facilitate assessments of local applicability of systematic reviews [5]. In addition, recent efforts have drawn on HSE to document the types of study designs included in reviews addressing health-system interventions [11]. To our knowledge, there have been no efforts to develop a comprehensive profile of the available research evidence addressing topics related to governance, financial and delivery arrangements within health systems, and implementation strategies that can support change in health systems, and the products derived from them.

## Methods

We drew on the global stock of the nine types of research evidence related to health systems contained in HSE as of July 2013. HSE draws its content from all of the major sources of each of the nine types of research evidence (e.g., Cochrane Library for systematic reviews of effects and protocols for such reviews, Economic Evaluation Database for economic evaluations, and Health Policy Monitor for health reform descriptions) [12],Unpublished data]. Each record contained in HSE is categorized according to a taxonomy of health system topics (e.g., remunerating providers) and domains (e.g., primary care), coded according to key features (e.g., systematic reviews are coded according to their methodological quality and the countries in which included studies were conducted) [13], and made available in seven languages. All of the eligibility assessments and coding are done by two independent raters. The methods underpinning the development and continuous updating of HSE are described in a separate manuscript [Unpublished data].

The data related to, and coding categories associated with, each of the records contained in HSE were used to calculate descriptive statistics that profile: 1) the distribution of research evidence, particularly systematic reviews, across health system topics and domains; 2) trends over time in how recently the literature was searched for systematic reviews and in the volume of publication for all types of research evidence; 3) the distribution of systematic reviews according to their methodological quality (assessed using AMSTAR (A MeaSurement Tool to Assess the quality of systematic Reviews) [13], as well as trends over time in the distribution of average quality scores; 4) the availability of user-friendly summaries (for systematic reviews) and links to additional content (for all types of research evidence) by source, type of systematic review, and language; and 5) the distribution of research evidence according to the type of low- and middle-income country (LMIC) focus. When summarizing our findings we often focus on systematic reviews, either because the data only apply to this type of research evidence (e.g., AMSTAR ratings) or because this type of research evidence is likely to be of greatest immediate relevance to health system policymakers and stakeholders.

## Results and Discussion

HSE contains (as of July 2013) 6,613 documents addressing topics related to health systems, which include:

• 140 review-derived products (94 evidence briefs and 46 overviews of systematic reviews);

• 2629 systematic reviews of effects

∘ 501 Cochrane reviews of which one in five have been produced by the Effective Practice and Organization of Care (EPOC) review group (111/501, 22%);

• 614 systematic reviews addressing other types of questions (e.g., reviews of observational studies that often assess the scale of problems or associations between variables and reviews of qualitative studies that often assess the nature of problems and/or how and why interventions work);

• 283 systematic reviews in-progress (i.e., systematic review protocols);

• 186 systematic reviews being planned (i.e., systematic review titles that have been registered and for which a protocol is being prepared);

• 1669 economic evaluations;

• 1092 health reform descriptions; and

• 209 descriptions of health systems.

### Topics and domains addressed

Most systematic reviews address topics related to delivery arrangements (n = 2,663) or implementation strategies (n = 1,653) in whole or in part, whereas much smaller numbers of systematic reviews address financial arrangements (n = 241) or governance arrangements (n = 231) (Table 1). Within the delivery arrangements category, ‘by whom care is provided’ (i.e., human resources) represents the largest sub-category (n = 1,426, of which 342 addressed multi-disciplinary teams). This is followed by “how care is designed to meet consumers’ needs” (n = 972, of which 562 addressed packages of care/care pathways/disease management) and “where care is provided” (n = 720, of which 427 addressed the site of service delivery). Within the implementation strategies category, consumer-targeted strategies represent the largest sub-category (n = 1,208), followed by provider-targeted strategies (n = 622) and organization-targeted strategies (n = 65). Within the governance arrangements category, organizational authority (i.e., what decisions can organizations like hospitals make and how) represents the largest sub-category (n = 89) and “consumer and stakeholder involvement” the second largest (n = 71). Within the financial arrangements category, remunerating providers (i.e., how providers are paid) represents the largest sub-category (n = 106), followed by “incentivizing consumers” (n = 85) and “financing systems” (i.e., how revenue is raised for health systems and services) (n = 56).

**Table 1:** **Number **(**and %**) **of documents addressing particular health system topics**, by type of document*

**Health system topics**	**All documents (n = 6,613)**		**Evidence briefs for policy (n = 94)**		**Overviews of systematic reviews (n = 46)**		Systematic reviews of effects** (n = 2,629)		**Systematic reviews addressing other types of questions (n = 614)**		**Systematic reviews in progress (n = 283)**		**Systematic reviews being planned (n = 186)**		**Economic evaluations (n = 1,669)**		**Health reform descriptions (n = 1,092)**	
**Governance arrangement**	1360	21%	46	49%	10	22%	117	4%	114	19%	15	5%	12	6%	80	5%	966	88%
• Policy authority	890	13%	24	26%	5	11%	26	1%	31	5%	8	3%	5	3%	23	1%	768	70%
• Organizational authority	648	10%	20	21%	4	9%	37	1%	52	8%	2	1%	3	2%	28	2%	502	46%
• Commercial authority	280	4%	8	9%	3	7%	17	1%	17	3%	1	0%	1	1%	9	1%	224	21%
• Professional authority	441	7%	16	17%	3	7%	33	1%	34	6%	2	1%	1	1%	25	1%	327	30%
• Consumer & stakeholder involvement	335	5%	21	22%	4	9%	35	1%	36	6%	4	1%	2	1%	6	0%	227	21%
**Financial arrangement**	1240	19%	47	50%	14	30%	171	7%	70	11%	23	8%	17	9%	153	9%	745	68%
• Financing systems	604	9%	22	23%	6	13%	32	1%	24	4%	6	2%	2	1%	23	1%	489	45%
• Funding organizations	230	3%	8	9%	4	9%	22	1%	9	1%	4	1%	0	0%	5	0%	178	16%
• Remunerating providers	400	6%	23	24%	11	24%	73	3%	33	5%	7	2%	1	1%	22	1%	230	21%
• Purchasing products & services	454	7%	9	10%	3	7%	20	1%	11	2%	3	1%	2	1%	91	5%	315	29%
• Incentivizing consumers	487	7%	16	17%	7	15%	72	3%	13	2%	10	4%	12	6%	46	3%	311	28%
**Delivery arrangement**	5590	85%	71	76%	40	87%	2145	82%	518	84%	225	80%	128	69%	1581	95%	882	81%
• How care is designed to meet consumers’ needs	2849	43%	32	34%	16	35%	808	31%	164	27%	84	30%	45	24%	1078	65%	622	57%
• By whom care is provided	2767	42%	58	62%	23	50%	1097	42%	329	54%	107	38%	59	32%	586	35%	508	47%
• Where care is provided	1677	25%	31	33%	13	28%	579	22%	141	23%	61	22%	33	18%	417	25%	402	37%
• With what supports is care provided	1792	27%	27	29%	23	50%	756	29%	152	25%	69	24%	25	13%	339	20%	401	37%
**Implementation strategy**	3031	46%	50	53%	25	54%	1425	54%	228	37%	145	51%	64	34%	633	38%	461	42%
• Consumer-targeted strategy	2297	35%	35	37%	16	35%	1078	41%	130	21%	114	40%	52	28%	489	29%	383	35%
• Provider-targeted strategy	1103	17%	38	40%	18	39%	502	19%	120	20%	34	12%	15	8%	203	12%	173	16%
• Organization-targeted strategy	104	2%	2	2%	5	11%	37	1%	28	5%	12	4%	2	1%	18	1%	0	0%

*This table does not provide data related to health system descriptions as they are not categorized in Health Systems Evidence according to health system topics. As a result, the total number of documents listed here is 6,613 but the total including the 209 health system descriptions indexed in Health Systems Evidence is 6,822.

**Systematic reviews of effects include 501 Cochrane reviews of which 111 (22%) have been produced by the Effective Practice and Organization of Care (EPOC) review group.

Many systematic reviews address particular diseases (n = 1,819), sectors (n = 1,634), providers (n = 1,582), and technologies (n = 667) (Table 2). Within the groupings of diseases indexed by HSE, the top five categories of diseases addressed by reviews are mental health and addictions (n = 962), cardiovascular disease (n = 343), diabetes (n = 276), maternal and child health (n = 284), and cancer (n = 222). While we found that approximately half (n = 1,582, 49%) of the systematic reviews in HSE address topics related to one or more types of providers, most within this grouping are focused on physicians (n = 945) and nurses (n = 692) and, to a lesser extent, allied health professionals (n = 391) as compared to pharmacists (n = 154) and lay/community health workers (n = 137). For the grouping of health system sectors, a relatively large number of reviews address primary care (n = 569), hospital care (n = 555), home care (n = 626), public health (n = 393), and rehabilitation (n = 195), but few address long-term care (n = 64). The majority of reviews addressing health system arrangements and implementation strategies related to the technologies category address drugs (n = 404) and surgery (n = 151) with far fewer addressing diagnostics (n = 90) and devices (n = 65).

**Table 2:** **Number** (**and %**) **of documents addressing particular domains**, by type of document*

**Domains**	**All documents (n = 6,613)**		**Evidence briefs for policy (n = 94)**		**Overviews of systematic reviews (n = 46)**		Systematic reviews of effects** (n = 2,629)		**Systematic reviews addressing other types of questions (n = 614)**		**Systematic reviews in progress (n = 283)**		**Systematic reviews being planned (n = 186)**		**Economic evaluations (n = 1,669)**		**Health reform descriptions (n = 1,092)**	
**Diseases**	3833	58%	49	52%	21	46%	1590	60%	229	37%	172	61%	83	45%	1279	77%	410	38%
*Infectious diseases*	808	12%	18	19%	4	9%	217	8%	35	6%	23	8%	22	12%	423	25%	66	6%
• HIV	295	4%	5	5%	2	4%	123	5%	19	3%	13	5%	16	9%	100	6%	17	2%
• Tuberculosis	99	1%	4	4%	2	4%	31	1%	9	1%	2	1%	2	1%	42	3%	7	1%
• Malaria	60	1%	8	9%	0	0%	11	0%	7	1%	7	2%	1	1%	26	2%	0	0%
• Diarrhoeal disease	52	1%	1	1%	0	0%	14	1%	0	0%	2	1%	1	1%	33	2%	1	0%
• Lower respiratory infections	71	1%	0	0%	0	0%	25	1%	2	0%	1	0%	2	1%	39	2%	2	0%
*Non-communicable diseases*	1884	28%	6	6%	9	20%	742	28%	111	18%	78	28%	30	16%	658	39%	250	23%
• Cancer	584	9%	3	3%	3	7%	183	7%	39	6%	18	6%	3	2%	209	13%	126	12%
• Cardiovascular disease	661	10%	2	2%	2	4%	306	12%	37	6%	26	9%	11	6%	204	12%	73	7%
• Diabetes	493	7%	2	2%	4	9%	234	9%	42	7%	14	5%	8	4%	119	7%	70	6%
• Alzheimer and other dementias	97	1%	1	1%	0	0%	46	2%	8	1%	7	2%	3	2%	12	1%	20	2%
• Chronic obstructive pulmonary disease	91	1%	1	1%	1	2%	40	2%	6	1%	7	2%	5	3%	24	1%	7	1%
*Other*	1833	28%	31	33%	13	28%	898	34%	129	21%	84	30%	37	20%	413	25%	228	21%
• Maternal and child health	678	10%	17	18%	5	11%	242	9%	42	7%	35	12%	12	6%	207	12%	118	11%
• Accidents	190	3%	1	1%	0	0%	84	3%	10	2%	8	3%	9	5%	52	3%	26	2%
• Mental health and addictions	1089	16%	15	16%	10	22%	637	24%	83	14%	42	15%	16	9%	155	9%	131	12%
**Technologies**	1805	27%	25	27%	9	20%	562	21%	105	17%	39	14%	15	8%	634	38%	416	38%
• Drugs	1047	16%	19	20%	9	20%	345	13%	59	10%	16	6%	11	6%	323	19%	265	24%
• Devices	174	3%	1	1%	0	0%	59	2%	6	1%	4	1%	1	1%	54	3%	49	4%
• Diagnostics	464	7%	2	2%	1	2%	76	3%	14	2%	9	3%	1	1%	200	12%	161	15%
• Surgery	379	6%	4	4%	0	0%	119	5%	32	5%	11	4%	3	2%	110	7%	100	9%
**Sectors**	3689	56%	44	47%	20	43%	1368	52%	266	43%	138	49%	51	27%	1025	61%	777	71%
• Primary care	1138	17%	23	24%	13	28%	460	17%	109	18%	38	13%	9	5%	206	12%	280	26%
• Home care	604	9%	6	6%	3	7%	287	11%	39	6%	26	9%	4	2%	134	8%	105	10%
• Hospital care	1537	23%	5	5%	3	7%	445	17%	110	18%	49	17%	24	13%	375	22%	526	48%
• Rehabilitation	345	5%	3	3%	0	0%	180	7%	15	2%	11	4%	3	2%	56	3%	77	7%
• Long-term care	252	4%	3	3%	3	7%	53	2%	11	2%	11	4%	3	2%	22	1%	146	13%
• Public health	1085	16%	20	21%	9	20%	349	13%	44	7%	47	17%	12	6%	395	24%	209	19%
**Providers**	3098	47%	33	35%	22	48%	1264	48%	318	52%	88	31%	41	22%	645	39%	687	63%
• Physician	1951	30%	14	15%	13	28%	767	29%	178	29%	50	18%	21	11%	345	21%	563	52%
• Nurse	1259	19%	17	18%	8	17%	526	20%	166	27%	40	14%	15	8%	284	17%	203	19%
• Pharmacist	338	5%	5	5%	4	9%	133	5%	21	3%	13	5%	7	4%	93	6%	62	6%
• Allied health professional	828	13%	9	10%	9	20%	334	13%	57	9%	27	10%	10	5%	170	10%	212	19%
• Lay/community health worker	322	5%	10	11%	3	7%	114	4%	23	4%	23	8%	8	4%	77	5%	64	6%

*This table does not provide data related to health system descriptions as they are not categorized in Health Systems Evidence according to topic domains. As a result, the total number of documents listed here is 6,613 but the total including the 209 health system descriptions indexed in Health Systems Evidence is 6,822.

### Trends in production over time

**Figure 1:**
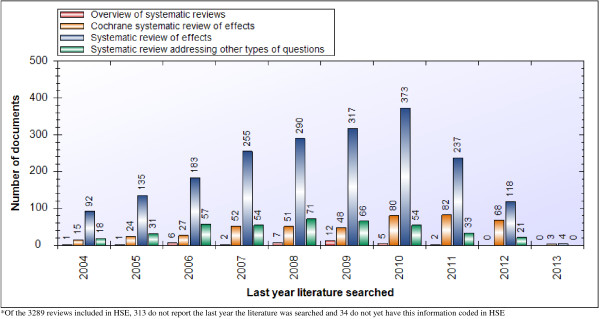
Last year literature was searched for systematic reviews and overviews of systematic reviews.

We found that 2,893 of the 3,243 systematic reviews (89%) reported the last year the literature was searched and, based on the available data, 1,584 (49%) were conducted within the last five years (i.e., since 2008) (Figure 1). Of the 350 reviews for which we do not have the last year the literature was searched, 199 (57%) were published within the last five years. Our sub-analysis of Cochrane reviews revealed that most of the 501 reviews (n = 493, 98%) reported the last year the literature was searched and 332 (66%) of these were conducted within the last five years, while only 153 (30%) were up to date within the last two years, which is the Cochrane Collaboration’s recommended timeframe for updating reviews [14]. For overviews of systematic reviews, 37 of 46 (80%) reported the last year the literature was searched, and 26 of these (56%) were conducted within the last five years (Figure 1).

**Figure 2:**
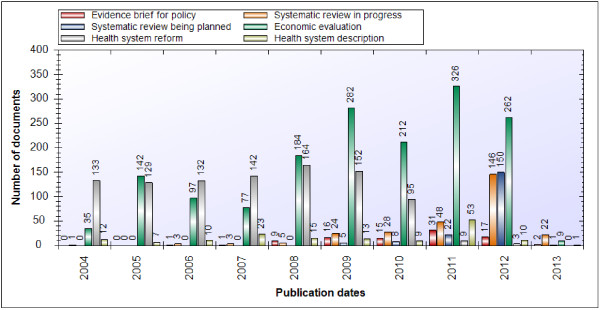
Publication dates for documents.

Regarding the year of publication, we found that 90 of the 94 (96%) evidence briefs for policy, 273 of the 283 (96%) systematic reviews in progress (i.e., protocols that had not yet been turned into a full review), all of the 186 systematic reviews being planned, 1,275 of the 1,669 (76%) economic evaluations (note that only those conducted in the last 10 years are indexed in HSE), 423 of the 1,092 (39%) descriptions of health reforms, and 101 of the 209 (48%) descriptions of health systems were published within the last five years (Figure 2).

### Methodological quality of systematic reviews

**Figure 3:**
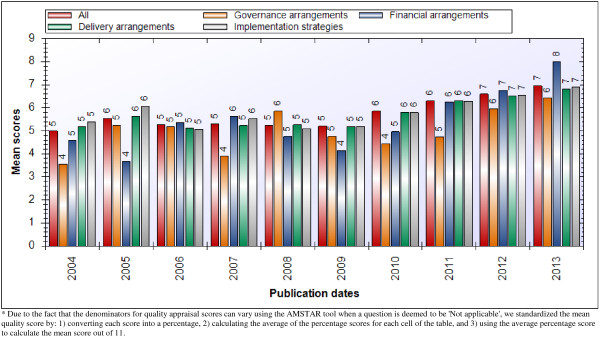
**Mean quality (AMSTAR) score.**

Most systematic reviews (n = 2,928, 90%) indexed in HSE have been quality appraised using AMSTAR and their methodological quality is (on average) moderate (i.e., between 4 and 7 out of 11), which has remained relatively consistent over time (Table 3 and Figure 3). The average methodological quality of reviews addressing delivery arrangements (6.3/11) and implementation strategies (6.3/11) was somewhat higher than those addressing financial (5.9/11) and governance arrangements (5.6/11). However, methodological quality differs substantially according to the type of systematic review. Those produced by the Cochrane Collaboration are of high quality (i.e., between 9 and 11 out of 11) across all second-level topic domains (with none falling outside this range). In contrast, systematic reviews of effects (which include Cochrane reviews) are of moderate quality (with no second-level category averages falling outside the range of 4.8–7.4) and quantitative reviews addressing other types of questions were low to moderate quality (with no averages falling outside the range of 3.6–5.4).

**Table 3:** Mean quality rating* of systematic reviews addressing particular health system topics, **by focus and type of systematic review**

Domains**	**All reviews (n = 2,928)†**	**All LMIC-focused reviews (n = 696)†**	**All types of systematic reviews†**		
			**Cochrane systematic reviews of effects (n = 484)†**	**Systematic reviews of effects (n = 2408)†**	(Quantitative) Systematic reviews addressing other types of questions*** (n = 520)†
**Governance arrangement (n = 197)**	5.6	5.4	10.3	6.6	4.5
• Policy authority (n = 46)	5.1	5.4	9.9	5.7	4.5
• Organizational authority (n = 69)	5.5	7.0	10.3	6.6	4.7
• Commercial authority (n = 33)	4.6	4.1	10.5	5.4	3.6
• Professional authority (n = 50)	5.4	4.8	10.3	6.4	4.3
• Consumer & stakeholder involvement (n = 60)	5.7	5.3	10.3	6.5	4.7
**Financial arrangement (n = 219)**	5.9	6.6	9.9	6.4	4.4
• Financing systems (n = 50)	5.1	5.7	10.5	6.1	3.7
• Funding organizations (n = 25)	4.7	5.5	9.3	5.0	3.5
• Remunerating providers (n = 97)	5.5	5.8	9.4	5.6	5.4
• Purchasing products & services (n = 27)	4.4	4.2	10.0	4.8	3.9
• Incentivizing consumers (n = 83)	7.0	7.7	10.0	7.4	4.5
**Delivery arrangement (n = 2,395)**	6.3	6.8	9.9	6.7	4.5
• How care is designed to meet consumers' needs (n = 883)	6.5	6.7	10.0	6.7	5.0
• By whom care is provided (n = 1,276)	6.2	6.6	9.9	6.6	4.5
• Where care is provided (n = 642)	6.6	7.3	9.9	7.0	4.9
• With what supports is care provided (n = 807)	6.1	6.8	10.0	6.4	4.5
**Implementation strategy (n = 1,519)**	6.3	6.8	9.8	6.5	4.9
• Consumer-targeted strategy (n = 1,118)	6.5	6.9	10.0	6.7	4.9
• Provider-targeted strategy (n = 562)	6.0	6.5	9.5	6.2	5.0
• Organization-targeted strategy (n = 53)	5.9	6.4	9.7	6.2	5.4

†The numbers refer to the number of reviews in each category with a completed quality appraisal. The total numbers for each column are as follows: All reviews = 3,243; All LMIC-focused reviews = 737; Cochrane systematic reviews of effects = 501; Systematic reviews of effects = 2,629; Systematic reviews addressing other types of questions = 614.

*Due to the fact that the denominators for quality appraisal scores can vary using the AMSTAR tool when a question is deemed to be 'Not applicable', we standardized the mean quality score by: 1) converting each score into a percentage, 2) calculating the average of the percentage scores for each cell of the table, and 3) using the average percentage score to calculate the mean score out of 11.

**The numbers beside each category refer to the number of quality appraisals available.

***This excludes systematic reviews of qualitative evidence that require a different approach to quality appraisal.

### Availability of user-friendly summaries and links to additional content

The majority (n = 2,168, 67%) of systematic reviews now have at least one user-friendly (English-language) summary available from one of the eight groups in the world that package, quality appraise and/or facilitate assessments of the local applicability of systematic reviews (Table 4). The largest producers of these structured decision-relevant summaries for reviews about health system arrangements are the DARE (n = 1,962, 90% of those with at least one summary) and the Cochrane Library through its plain language summaries of reviews (n = 505, 23%). However, these review summaries are not targeted specifically at health system policymakers and stakeholders. The three groups representing the next largest shares of summaries of reviews about health system arrangements – Rx for Change (n = 211, 10%), Health Evidence™ (n = 231, 11%), and Australasian Cochrane Centre (n = 85, 4%) – are more targeted at these groups. A number of reviews also have one or more summaries available in Spanish (n = 474) and French (n = 391), but fewer summaries are available in the four remaining languages (14 for each of Arabic and Russian, 36 for Chinese and 1 for Portuguese).

**Table 4:** **Number **(**and %**) **of user**-**friendly summaries available for systematic reviews**, by type of systematic review*

**Source of summary**	All reviews (n = 3,243)**		**Systematic reviews of effects (n = 2,629)**		**Systematic reviews addressing other types of questions (n = 614)**	
Australasian Cochrane Centre Policy Liaison Initiative	85	3%	85	3%	0	0%
Cochrane Library (plain language summaries)	505	16%	504	19%	1	0%
Database of Review of Effects	1,962	60%	1,821	69%	141	23%
Effective Health Care Research Programme Consortium	14	0%	14	1%	0	0%
Health Evidence™	231	7%	228	9%	3	0%
Reproductive Health Library	21	1%	21	1%	0	0%
Rx for Change	211	7%	201	8%	10	2%
SUPPORT	34	1%	34	1%	0	0%

*This table only provides counts of links available in English.

**1,075 systematic reviews have no independently produced user-friendly summary.

Across all documents contained in HSE (n = 6,613), more than half (n = 3,893, 59%) have no links to one or more independently produced structured decision-relevant summaries (Table 5). Most documents have links to a scientific abstract from PubMed (n = 4,549), the Cochrane Library (n = 1,994, of which 299 have been translated into Spanish and 28 have been translated into French) or from another publisher (n = 4,281, of which 55 have been translated into French). Two thirds of the documents have a link to a full-text report that can be accessed without a subscription (n = 4,638, 70%); however, countries that have a national license to the Cochrane Library can access full Cochrane reviews (n = 501) and protocols of Cochrane reviews (n = 283) for free as well.

**Table 5:** **Number of links available for all types of document**, by language*

**Links**	**Language**						
	**English**	**Arabic**	**Chinese**	**French**	**Portuguese**	**Russian**	**Spanish**
**No. of user-friendly summaries**							
• 0	3,893	7,855	7,835	7,479	7,868	7,855	7,411
• 1	3,260	14	32	375	1	14	438
• 2	507	0	2	15	0	0	19
• 3	141	0	0	0	0	0	1
• 4	44	0	0	0	0	0	0
• 5+	24	0	0	0	0	0	0
**Abstracts**							
• PubMed	4,549	0	0	0	0	0	0
• Cochrane Library	1,994	0	0	28	0	0	299
• Publisher	4,281	0	0	55	0	0	0
**Full-text**	4,638	63	63	731	2	62	152

*Includes IberoAmerican (for Spanish) and BVS Salud (for Portuguese).

### Low- and middle-income country (LMIC) focus

We found 1,228 (19%) documents in HSE that have an LMIC focus (Table 6), half of which are systematic reviews of effects (n = 614, 50%) and a lower percentage of which are economic evaluations (n = 280, 23%), systematic reviews addressing other types of questions (n = 123, 10%), and health system descriptions (n = 92, 7%). The 737 systematic reviews with an LMIC focus (which are the subset of documents described in Table 6 that we focus on here) were most often the result of including at least one study conducted in an LMIC setting (n = 712, 97%) with far fewer identified as having an LMIC setting as the target of the document (n = 193, 26%) or having at least one author from an LMIC (n = 118, 16%).

**Table 6:** **Number** (**and %**) **of documents addressing health system topics**, **by type of low**- **and middle**-**income country** (**LMIC**) **focus**

**Health system topics**	**Type of LMIC focus**							
	Any category* (n = 1,228)		**Target of document (n = 648)**		**At least one LMIC author (n = 392)**		**At least one LMIC study included (n = 781)**	
**Governance arrangement**	117	10%	77	12%	46	12%	84	11%
• Policy authority	57	5%	46	7%	25	6%	40	5%
• Organizational authority	36	3%	25	4%	14	4%	29	4%
• Commercial authority	18	1%	16	2%	7	2%	14	2%
• Professional authority	26	2%	13	2%	6	2%	22	3%
• Consumer & stakeholder involvement	25	2%	15	2%	12	3%	19	2%
**Financial arrangement**	166	14%	120	19%	82	21%	108	14%
• Financing systems	62	5%	51	8%	25	6%	46	6%
• Funding organizations	18	1%	12	2%	5	1%	14	2%
• Remunerating providers	51	4%	33	5%	22	6%	42	5%
• Purchasing products & services	42	3%	33	5%	27	7%	14	2%
• Incentivizing consumers	59	5%	44	7%	27	7%	41	5%
**Delivery arrangement**	944	77%	472	73%	341	87%	618	79%
• How care is designed to meet consumers' needs	485	39%	318	49%	220	56%	248	32%
• By whom care is provided	445	36%	180	28%	141	36%	340	44%
• Where care is provided	287	23%	165	25%	120	31%	193	25%
• With what supports is care provided	202	16%	52	8%	40	10%	168	22%
**Implementation strategy**	516	42%	207	32%	154	39%	407	52%
• Consumer-targeted strategy	390	32%	151	23%	112	29%	318	41%
• Provider-targeted strategy	195	16%	85	13%	63	16%	147	19%
• Organization-targeted strategy	17	1%	7	1%	3	1%	12	2%

*Of these documents, 737 are systematic reviews.

Similar to the full set of reviews contained in HSE, the systematic reviews with an LMIC focus most often addressed topics related to delivery arrangements (n = 590) and implementation strategies (n = 394) in whole or in part, with far fewer addressing financial (n = 74) and governance arrangements (n = 55). The most common sub-categories under delivery arrangements containing systematic reviews with an LMIC focus were “by whom is care provided” (n = 319), “how care is designed to meet consumers’ needs” (n = 236), “where care is provided” (n = 171), and “with what supports is care provided” (n = 161). The majority of systematic reviews within the implementation strategies category address consumer-targeted strategies (n = 310), while fewer addressing provider-targeted strategies (n = 132). Within the governance arrangements category, policy authority (n = 20) represents the largest sub-category and within the financial arrangements category, “incentivizing consumers” (n = 29), “remunerating providers” (n = 27) and “financing systems” (n = 26) represent the largest sub-categories.

**Figure 4:**
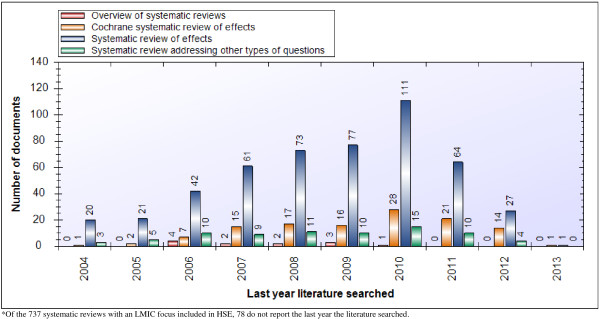
Last year literature was searched for systematic reviews and overviews of systematic reviews with an LMIC focus.

The systematic reviews identified as having an LMIC focus also have a similar profile to the global stock of reviews in terms of how recently they were conducted and their quality. As outlined in Figure 4, slightly more than half of the reviews (n = 403, 54%) were conducted within the last five years (since 2008) and of the 12 overviews of systematic reviews with an LMIC focus 6 (50%) were conducted within the last five years. In addition, we found that all of the systematic reviews in progress (n = 25), systematic reviews being planned (n = 17), and health reform descriptions (n = 3), as well as 237 of 280 (85%) economic evaluations, 58 of 60 (97%) evidence briefs for policy and 42 of 92 (46%) health system descriptions, with an LMIC focus, were published within the last five years. In terms of quality, almost all of the reviews with an LMIC focus (n = 696, 94%) have been appraised for methodological quality using AMSTAR and (on average) have a moderate quality score (range = 4.2–7.7 out of a possible score of 11) across topic domains (Table 3).

## Conclusions

We have provided a detailed examination of the global stock of research evidence relevant to health systems policymaking. Systematic reviews of effects are the most common of the nine types of research evidence in HSE (n = 2,629, 40%). Most systematic reviews address topics related to delivery arrangements (n = 2,663) or implementation strategies (n = 1,653) in whole or in part, which may reflect a tendency among more clinically oriented researchers to first examine the effects of an intervention and then (faced with feasibility and other concerns raised by policymakers and stakeholders) to examine the effects of alternative ways of delivering the intervention or implementing it on a large scale. Half (49%) of the systematic reviews presented findings based on searches conducted within the last five years (since 2008), quality ratings ranged from 5.6 (for governance arrangements) to 6.3 (for delivery arrangements and implementation strategies) out of a possible 11, nearly one in four reviews (n = 737, 23%) contain at least one study from a LMIC, and 1,075 reviews have no independently produced structured decision-relevant summary.

This study has one key strength and one potential limitation. Our analysis represents the first effort (at least to our knowledge) to systematically assess the global stock of nine types of research evidence relevant to health systems policymaking. Moreover, we have developed an approach to real-time reporting for all of the tables and figures included in our analysis, which will allow us to update the tables and figures at any time and identify changes over time. The potential limitation of our study is that, by relying on one data source for our analysis, we have missed including relevant systematic reviews. However, we believe this possibility to be remote given that HSE systematically culls relevant documents from a number of databases (Medline, Cochrane Database of Systematic Reviews, three databases – DARE, PROSPERO, Economic Evaluations Database – from the Centre for Reviews and Dissemination, Rx for Change, and the Cochrane Qualitative and Implementation Methods Group’s reference database for qualitative reviews) and many other sources such as listservs (e.g., EvidenceUpdates and PAHO EQUIDAD) and websites (e.g., AHRQ, Campbell Collaboration, EPPI-Centre and several more). As a result, searching other sources would be unlikely to identify many documents. For those interested in reviewing the full list of sources and methods used to build and continuously update HSE we provide these details in a separate manuscript [Unpublished data].

Based on the profile of research evidence provided here, particularly the profile of systematic reviews, we believe that greater effort should be placed on several priorities to enhance the usefulness of research evidence about health systems and support its use by policymakers and stakeholders. First, there is a need to support efforts to regularly update systematic reviews, as doing so will ensure that policymakers and stakeholders have access to the most recent synthesized research evidence. As outlined in our findings, half of the systematic reviews in HSE were conducted five or more years ago and a large proportion of those produced by the Cochrane Collaboration have also not been updated within their recommended timeline of two years. Such efforts could be supported (at least partially) by funders of systematic reviews providing resources both for new systematic reviews and for updating existing reviews (e.g., by identifying reviews that address timely policy questions but are out of date).

Second, there is a need to increase the average quality of systematic reviews. This is particularly evident for systematic reviews addressing questions other than effectiveness and/or for those addressing topics related to governance and financial arrangements (many of which are reviews addressing other types of questions), which generally scored lower for quality as compared to reviews addressing questions of effectiveness. Registering titles and protocols for systematic reviews and implementing specific quality standards as part of the registration process is a promising mechanism that may help increase the overall quality of reviews. This requirement from the Cochrane Collaboration contributes (at least in part) to the average level of quality for Cochrane reviews being significantly higher than the rest of the reviews in HSE. In addition, PROSPERO [15] now offers an international prospective register of systematic reviews requiring minimum methodological standards based on the PRISMA statement [16]. These efforts could be further supported by journals requiring systematic reviews to be registered in order to be considered for publication.

It should be noted that the AMSTAR tool was designed originally for reviews of effects. However, the fundamental methodological requirements for reviews that include quantitative evidence remain the same and where they differ there is an option to mark a question as “not applicable” to the score. As noted in our results, we did not conduct quality appraisals of reviews that draw exclusively on qualitative research evidence given that there is currently no quality appraisal tool available (at least to our knowledge) that takes into account the unique methods and approaches to data analysis used in these types of reviews (although two of us – MGW and JNL – are currently finalizing a tool that will be able to be used for this purpose).

Third, there is a need to use the global stock of systematic reviews to start developing global guidance to support evidence-informed policies about health systems [17-19]. However, the utility of such guidelines is dependent upon the availability of systematic reviews addressing the full range topics related to governance, financial and delivery arrangements within health systems, and about implementation strategies that can support change in health systems. This will require diversifying the types of topics addressed in systematic reviews (particularly for governance and financial arrangements or for increasingly important topic-specific domains such as long-term care, which are relatively underserved in the current global stock) as well as ensuring relevance to LMICs (for which there are comparatively few reviews). Addressing this could also include conducting assessments of whether the current distribution of systematic reviews corresponds to policymakers’ and stakeholders’ priorities.

## Abbreviations

DARE: Database of abstracts of reviews of effects; HSE: Health systems evidence; LMICs: Low- and middle-income countries.

## Competing interests

John Lavis led the creation and oversees the continuous updating of Health Systems Evidence (http://www.healthsystemsevidence.org). Michael Wilson also helped lead the creation of Health Systems Evidence and contributes to its continuous updating. Kaelan Moat coordinates the continuous updating of Health Systems Evidence.

## Authors’ contributions

All authors contributed to the conception and design of the study; MGW and JNL drafted the original manuscript; MGW conducted the data analysis; and KM reviewed, revised and conducted a quality check of all data analyses. All authors reviewed and approved the final version of the manuscript.
